# VEGFR-2: One of Pioglitazone’s Signaling Pathways in the Heart

**DOI:** 10.5935/abc.20180147

**Published:** 2018-08

**Authors:** Marcos Ferreira Minicucci, Leonardo Antonio Mamede Zornoff

**Affiliations:** Departamento de Clínica Médica - Faculdade de Medicina de Botucatu - Universidade Estadual Paulista Júlio de Mesquista Filho-UNESP, Botucatu, SP – Brazil

**Keywords:** Peroxisome Proliferators/adverse effects, Glucose/metabolism, Lipids/metabolism, Pioglitazone, Diabetes Mellitus/drug therapy

Pioglitazone is currently the only commercially available hypoglycemic agent that improves insulin sensitivity. Its mechanism of action involves activation of peroxisome proliferator-activated receptor (PPAR) gamma, a nuclear receptor that alters the transcription of genes involved in glucose and lipid metabolism and in energy balance.^1^^,^^2^ Hence, pioglitazone increases insulin sensitivity, reduces glucose production by the liver and increases glucose uptake by peripheral tissues.^1^^,^^2^

Beneficial effects of pioglitazone include a low risk of hypoglycemia and the improvement of cardiovascular risk factors such as lipid profile and endothelial function.^1^^,^^2^ The main side effects of the drug include weight gain, especially due to the risk of edema or heart failure, increased risk of bone fractures and its association with prostate cancer, which has been questioned in recent studies.^2^ Pioglitazone is relatively potent in reducing glycated hemoglobin A1c levels; however, previous studies have shown no benefit in performing a more intensive control of glucose on cardiovascular mortality as compared with a less intensive control.^3^ This is important since cardiovascular diseases are still the most common causes of diabetes.^3^

Also, recent studies have reported beneficial effects of sodium-glucose transporter 2 (SGLT2) inhibitors and glucagon-like peptide-1 (GLP-1) analogues in the secondary prevention of cardiovascular events.^4^^-^^7^ For this reason, these should be the drugs of choice to be used in combination with metformin in patients with established cardiovascular diseases according to the American Diabetes Association recommendations.^8^ Nevertheless, few studies have been conducted on patients with recently diagnosed diabetes and low prevalence of cardiovascular diseases.

In this context, the TOSCA.IT study compared the cardiovascular effects of the addition of pioglitazone or sulfonylureas to metformin in patients with type 2 diabetes.^9^ The study showed that, in absence of clinically evident cardiovascular disease, both treatments are suitable options. However, considering the long-term metabolic effects, pioglitazone plus metformin may be considered the therapy of choice, since this was associated with a lower risk for hypoglycemia and a reduction in cardiovascular events by nearly 30%.^9^ These findings agree with the beneficial effects of pioglitazone on cardiovascular events reported in the PROactive and PERISCOPE studies.^10^^,^^11^

With respect to potential pathophysiological mechanisms of the cardiovascular benefits of pioglitazone, it is believed that, in addition to its metabolic effect in reducing insulin resistance, this thiazolidinedione may have a direct effect on the heart. Experimental studies have already reported the effects of pioglitazone in fibrosis, apoptosis and myocardial hypertrophy.^12^^-^^14^ In this issue of *Arquivos Brasileiros de Cardiologia*, Zhong et al.^15^ investigated whether the effects of pioglitazone on cardiomyocyte apoptosis and hypertrophy occur via vascular endothelial growth factor receptor-2 (VEGFR-2) signaling. VEGFR-2 is a tyrosine kinase receptor that activates intracellular signaling pathways involved in cell proliferation, migration and cycle. First, using the reverse pharmacophore mapping technique, the authors identified VEGFR-2 as the best-ranked potential target for pioglitazone. Then, the authors isolated cardiomyocytes from Sprague-Dawley rats and evaluated the effects of pioglitazone and the VEGFR-2-selective inhibitor apatinib on two outcomes – cardiomyocyte apoptotic rate using flow cytometry and hypertrophy using [^3^H]-leucine incorporation. Interestingly, the results showed a reduction not only in cardiomyocyte viability but also in cardiomyocyte hypertrophy induced by angiotensin II *in vitro*. Besides, both pioglitazone and apatinab increased the expression of Bax and phosphorylated P53 and decreased the expression of phosphorylated VEGFR-2, Akt, and mTOR in the cardiomyocytes. Studies in the literature are controversial regarding the effects of pioglitazone on cardiomyocyte hypertrophy and apoptosis,^12^^-^^14^ maybe due to different dosages and models used in the studies. However, in the study in question, the authors suggested that heart failure patients would not benefit from therapy with pioglitazone, since although it attenuated cardiomyocyte hypertrophy, the drug induced apoptosis of these cells.

In addition, although the direct effects of pioglitazone on the heart are still under investigation, Zhong et al.^15^ make an important contribution to the field, as suggesting that one of the mechanism of action of pioglitazone is via VEGFR-2. Also, if we consider that there is clinical evidence of the beneficial effects of this hypoglycemic agent on cardiovascular outcomes, further studies should be conducted to better define the role of pioglitazone in cardiovascular diseases in diabetics.
